# Structural and Optical Characterizations of Cadmium Chalcogenide Layers on Polyamide Formed Using Monotelluropentathionic Acid

**DOI:** 10.3390/ma15030787

**Published:** 2022-01-20

**Authors:** Remigijus Ivanauskas, Linas Samardokas, Judita Sukyte, Skirma Zalenkiene, Ingrida Ancutiene

**Affiliations:** Faculty of Chemical Technology, Department of Physical and Inorganic Chemistry, Kaunas University of Technology, 50254 Kaunas, Lithuania; linas.samardokas@ktu.lt (L.S.); vjsukyte@gmail.com (J.S.); skirma.zalenkiene@ktu.lt (S.Z.); ingrida.ancutiene@ktu.lt (I.A.)

**Keywords:** monotelluropentathionic acid, thin layers, surface properties, functional materials

## Abstract

Mixed cadmium tellurides–cadmium sulfide thin layers were formed on the polyamide PA 6. Monotelluropentathionic acid (H_2_TeS_4_O_6_) was used as a precursor of tellurium and sulfur. A low-temperature, nontoxic, and cost-effective SILAR method was applied. Cadmium telluride (CdTe) and sulfide (CdS) layers were formed through the consecutive reactions of sorbed/diffused chalcogens species from telluropentathionate anion (TeS_4_O_6_^2−^) with functional groups of polyamide and alkaline cadmium sulfate. The pseudo-second-order rate and Elovich kinetic models were the best fit to quantify an uptake of chalcogens and cadmium on PA 6. The effects of chalcogens and Cd on the structure and optical properties of PA 6 were characterized using UV-Vis and IR spectra. The clear changes of these properties depended on the concentration and exposure time in the precursor solutions. Fourier transform infrared spectroscopy and ultraviolet-visible spectroscopy were applied in order to evaluate the effect of the chalcogen species on the changes in structure of polyamide 6 films, depending on the exposure time in the solution of the chalcogens precursor and its concentration. The optical bandgap energy of the formed layers was found to be in the order of 1.52–2.36 eV. Studies by scanning electron microscopy and atomic force microscopy reveal that the diameter of the average grain is approximately 30 nm. The grains are conical in shape and unevenly distributed all over the surface of the substrate.

## 1. Introduction

Compounds of the II–VI group of elements or semiconductors based on these compounds are important luminescent, lasing, and optoelectronic materials [1]. Due to the successful synthesis of semiconductor compounds of type II–VI, these materials can be widely used for mechanical and optoelectronic purposes; therefore, further study of such semiconductors is extremely important [1,2,3]. Due to its high optical absorption coefficient (>10^4^ cm^−1^) and optimal bandgap (1.5 eV), CdTe is an efficient photoconverter [2]. Therefore, it is one of the main widely used photovoltaic materials for the successful development of high-efficiency solar cells and modules.

Cadmium chalcogenides CdE (E = S, Te), e.g., cadmium sulfide, and telluride thin films are used in laser windows and photo-electric cells, photothermal conversion, and solar cells, etc. [3,4]. With the aim of low-cost solar cells having thin films, common preliminary materials are employed, which are used in the semiconductor industry [5,6]. CdTe and CdS are typical layered semiconductors with almost complete chemical bonds inside the layer and, because of this, they have a low density of dangling bonds on the surface [7,8] and mechanical weakness due to the weak Van der Waals forces between layers. It has also been found that the solar cell efficiency correlated with the chemical and morphological properties of the surfaces [9].

A wide range of methods have been applied to produce cadmium chalcogenide thin layers, including electrodeposition [10], high-frequency magnetron sputtering [11], spray pyrolysis [12], thermal co-evaporation [13], the thermal decomposition of cadmium organic precursors [14], vapor deposition [15], electron beam evaporation [16], and others. Unfortunately, most of these techniques require special equipment and conditions such as high temperature, vacuum, and conductive substrates, and they usually consist of many stages, making them unsuitable and costly for the manufacture of the majority of polymers.

Only chemical methods used in the formation process of cadmium chalcogenide thin layers allow the development of good quality thin layers through the application of low temperature conditions. Layers of chalcogenides of metals of the I–II B groups on the substrate of an amphiphilic polyamide 6 (PA 6) were successfully obtained by the wet chemical method of adsorption–diffusion technology [17,18,19,20,21]. Very similar to this method, the successive ionic layer adsorption and reaction (SILAR) technique of the wet chemical methods is widely used to form these layers on a glass substrate [22,23,24]. In any case, due to its efficiency and economy, the SILAR method is one of the most promising for obtaining high-quality thin layers of chalcogenides on polymers for photovoltaic applications. In this method, the formation of metal chalcogenide layers takes place when the polymer substrate is exposed to a solution of the chalcogens precursor and further in a solution of metal ions with a suitable complexing agent. The production of thin-layer solar cells on polymer substrates or flexible metal foils offers several advantages for both space and terrestrial applications. The thickness of the solar cell is only 5 µm, while the thickness of the flexible substrate is 5–10 µm, so the weight savings compared to glass substrates are significant.

This work evaluated more closely what effect chalcogens have on the properties of semiconductors, along with their suitability for photovoltaic application. The selection of suitable precursors and their studies illustrate how chalcogens’ compounds could be used in electroactive materials. The preparation of chalcogen layers using chalcopolythionic acids, such as monoselenopentathionic H_2_SeS_4_O_6_ and monotelluropentathionic H_2_TeS_4_O_6_, has caught the attention of researchers [20,25,26]. These precursors are more beneficial than conventional sources due to their higher stability. It was determined that, under our experimental conditions, the solution of monotelluropentathionic acid was more stable than the alkaline metal salt solution of a similar concentration [26] used in our previous experiment.

## 2. Materials and Methods

### 2.1. Materials

Tellurous acid (98% purity), sulfuric acid (95%), acetic acid (99%), and ammonia water (25%) were acquired from Labochema (Vilnius, Lithuania) Na_2_S_2_O_3_ × 5H_2_O (99.5% purity); barium perchlorate (97% purity) and CdSO_4_ × 8/3H_2_O (99% purity) were purchased from SigmaAldrich, Saint Louis, MO, USA. Film of polyamide 6 (PA 6) (specification TU 6-05-1775-76) and grade PK-4 (Nylon 6) was used as a substrate. The main characteristics of Polyamide 6 (Nylon 6) are presented in Table 1.

### 2.2. Cadmium Chalcogenides Layer Formation Materials

**Figure 1:**
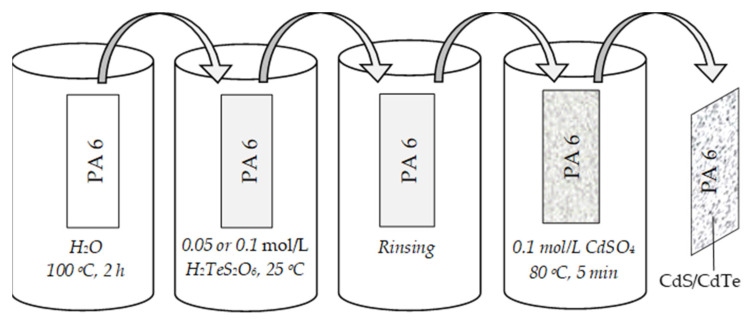
Schematics of cadmium chalcogenide layer formation procedure.

Films of polyamide 6, 15 mm × 70 mm in size and 70 μm in thickness, were used. The porosity of the polymer was determined by the *β*-method on a Quantasorb (Osaka, Japan). The pore size of PA 6 was found to be much less than 1.5 nm, indicating that it is practically non-porous [27]. Prior to chalcogenization, the samples of polyamide were boiled in distilled water at 100 °C for 2 h to remove the remaining un-polymerized monomer residues and dried in the desiccator over dehydrated CaCl_2_ for 24 h. Then, the samples of PA 6 were used in the analysis and in further experiments. The formation scheme of cadmium chalcogenide layers is shown in Figure 1.

Firstly, the samples of polyamide film were chalcogenized at 25 °C in aqueous solutions of 0.05 and 0.1 mol/L monotelluropentathionic acid. The experimental conditions are shown in Table 2.

Sodium telluropentathionate, Na_2_TeS_4_O_6_, was synthesized according to the procedure [28]:

H_2_TeO_3_ + 4Na_2_S_2_O_3_ + 4CH_3_COOH → Na_2_TeS_4_O_6_ + Na_2_S_4_O_6_ + 4CH_3_COONa + 3H_2_O (1)

Then, BaTeS_4_O_6_ was produced using barium perchlorate:

Na_2_TeS_4_O_6_ + Ba(ClO_4_)_2_ → BaTeS_4_O_6_ + 2NaClO_4_ (2)

H_2_TeS_4_O_6_ acid was isolated from synthesized barium salt in the reaction of BaSO_4_ precipitation with the solution of 3 mol/L sulphuric acid:

BaTeS_4_O_6_ + H_2_SO_4_ → H_2_TeS_4_O_6_ + BaSO_4_ (3)

Next, to form mixed cadmium chalcogenide layers, chalcogenized samples of PA 6 were treated with an alkaline (pH was found to be 11.5) cadmium salt solution at 80 °C for 5 min. An amount of 0.1 mol/L CdSO_4_ solution was made from crystalline CdSO_4_ × 8/3H_2_O; the pH of this solution was adjusted using a solution of ammonia. A complex cation was formed according to the reaction (4):

Cd^2+^ + 4NH_3_ × H_2_O → [Cd(H_2_O)_2_(NH_3_)_4_]^2+^ + 2H_2_O (4)

After treatment in a cadmium salt solution, the samples were washed in distilled water, dried in a desiccator over CaCl_2_ for 24 h, and analyzed.

### 2.3. Instrumental Analysis

The UV-Vis (190–800 nm) and IR (400–1400 cm^−1^) spectra were recorded by a spectrophotometer Spectronic**^®^** Genesys^TM^ 8 (Spectronic instruments, Runcorn, UK) and spectrometer Perkin Elmer GX system FTIR (Thermo Fisher Scientific, Waltham, MA, USA), respectively.

A D8 Advance diffractometer (Bruker AXS, Karlsruhe, Germany) was used for X-ray diffraction analysis of the deposited layers of cadmium chalcogenide. Operating voltage and current in the tubes of the device were 40 kV and 40 mA. To record diffraction patterns in the Bragg–Brentano geometry, a fast counting one-dimensional Bruker LynxEye detector (Bruker AXS, Karlsruhe, Germany) based on silicon strip technology was used. The X-ray was filtered through a 0.02 mm Ni filter to suppress Cu-k alpha radiation, and the samples were scanned in the 2*θ* = 3–70° range at a scan rate of 6° 1/min. The diffractometer is supplied together with the software package DIFFRAC.SUITE^TM^. X-ray diffractograms of deposited layers were processed using the software packages Crystallographica Search-Match v. 2.1, ConvX v.1.0 and Microsoft Office Excel 2013.

To obtain infrared spectra in the range of 400–1400 cm^−1^, a Perkin-Elmer FTIR Spectrum GX spectrometer (Thermo Fisher Scientific, Waltham, MA, USA) was used.

The thickness of the layers was measured using the scanning electron microscopy (SEM) images of the cross-section. A sample cross section was prepared by cutting the layers of Cd*_x_*S*_y_*–Cd*_x_*Te*_y_*on PA 6 with a sharp razorblade and was mounted for imaging using carbon tape. SEM imaging was performed with a Scanning Electron Microscope Quanta 200 FEG (FEI, Eindhoven, The Netherlands). The microscope was also equipped with an Energy Dispersive X-ray Spectrometer (EDS) detector XFlash 4030 (Bruker AXS Microanalysis, Berlin, Germany).

The quantitative microscopic studies of the surface roughness and the morphology of the formed layers of cadmium chalcogenide on the PA 6 were carried out using a QUESANT QScope-250 atomic force microscope (AFM) (Quesant Instrument Corporation, Agoura Hills, CA, USA). The dry samples were investigated in contact mode using commercial (Nano Technology Instruments—Europe BV) Si cantilevers CSG10 series with a force constant k = 0.2 Nm^−1^ and tip curvature of 10 nm. The images were analyzed, and image metrology was made using SPIP (Image Metrology, A/S, Lyngby, Denmark) and Quesant Corp. software v. 2.3.

A Perkin-Elmer 503 atomic absorption spectrometer was used for determination of the total amount of cadmium (λ = 228.8 nm) and tellurium (λ = 214.3 nm) in the cadmium chalcogenides layers. The sensitivity of atomic adsorption spectroscopic determination for Te and Cd, respectively, is 1.0 μg/cm^3^ and 0.025 μg/cm^3^ for 1% absorption.

The concentration of sulfur in PA 6 as sulfate was measured using the turbidimetric method [29] with a photoelectric colorimeter KFK-4 (Zagorsk Optical-Mechanical Plant, Sergiyev Posad, Russia) at λ = 400.0 nm. SO_4_^2−^ in the concentration range of 1–15 mg/dm^3^ can be determined by reaction with BaCl_2_ in the form of BaSO_4_. The deviation in the concentration interval of 5−10 mg/dm^3^ is 8%.

### 2.4. Modeling the Kinetics of Sorption

In order to quantify the extent of uptake in adsorption kinetics, six simple adsorption reaction and adsorption diffusion models were tested. Lagergren’s pseudo first-order rate equation is expressed as follows [30]:

(5) $$ln\left(q_{e}-q_{t}\right)=lnq_{e}-k_{1}t$$

where *q_t_* is the adsorption capacity (μmol/cm^2^) at time *t* (h), *q_e_* is the adsorption capacity (μmol/cm^2^) at equilibrium time (48 h), and *k*_1_ is the rate constant of the pseudo first–order adsorbtion (h^−1^). The values of *k*_1_ and *q_e_* can be obtained from the intercept and slope of the plot of *ln*(*q_e_*_−_ *q_t_*) versus *t*.

A pseudo second-order adsorbtion kinetic rate equation (Ho’s equation) is [31]:

(6) $$\frac{t}{q_{t}}=\frac{1}{k_{2}q_{e}^{2}}+\frac{t}{q_{e}}$$

where *k*_2_ is the rate constant of the pseudo second-order adsorbtion (cm^2^/μmol·h). The values of *k*_2_ and *q_e_* were obtained from the intercept and slope of the plot of *t·q_t_^−^*^1^ versus *t*.

The integrated form of Elovich’s equation becomes [32]:

(7) $$q_{t}=\frac{1}{b}ln\left(t+t_{0}\right)-\frac{1}{b}lnt_{0}$$

(8) $$t_{0}=\frac{1}{ab}$$

where *a* (μmol/cm^2^·h) is the initial adsorption rate. The constant a is regarded as the initial rate because (d*q_t_*/d*t*) approaches a when *q_t_* approaches 0 [33]*; b* (cm^2^/μmol) is the desorption constant. With the assumption of *t* >> *t*_0_ (this will be justified later), this is simplified as:

(9) $$q_{t}=\frac{1}{b}ln\left(ab\right)+\frac{1}{b}ln\left(t\right)$$

The values of *a* and *b* can be obtained from the slope and intercept of the plot of *q_t_* versus *lnt*. The film diffusion mass transfer rate equation presented by Boyd et al. [34] is:

(10) $$ln\left(1-\frac{q_{t}}{q_{e}}\right)=-Rt$$

where *R* is the liquid film diffusion constant (h^−1^). A plot of *ln*(1 − *q_t_*/*q_e_*) versus *t* should be a straight line with a slope −*R* if the film diffusion is the rate limiting step.

The Weber–Morris intraparticle diffusion model is [35]:

(11) $$q_{t}=k_{int}\sqrt{t}$$

where *k_int_* is intraparticle diffusion rate constant (μmol/cm^2^·h^−1/2^). A plot of *q_t_* versus *t*^1/2^ should be a straight line with a slope *k_int_* when the intraparticle diffusion is a rate-limiting step. Dumwald–Wagner proposed another intraparticle diffusion model as [36]:

(12) $$ln\left(1-{\left(\frac{q_{t}}{q_{e}}\right)}^{2}\right)=-Kt$$

where *K* is the Dumwald–Wagner rate constant of adsorption (h^−1^). A plot of *ln*(1 − (*q_t_/q_e_*)^2^) versus *t* should be linear, and the rate constant *K* can be obtained from the slope.

The experimental *q_t_* values are first processed by linearized equations to determine the model parameters, and, if *r*^2^ > 0.95, the determined values were used to reconstruct the kinetic equations. The kinetic curves show an overlap of experimental results (points). The linear correlation coefficients (*r*^2^) showed the correspondence of the experimental data to the linearized forms of the kinetic equations. The average percentage errors (APE) indicate the agreement between the experimental and predicted adsorption capacity values used to construct the kinetic curves [37]:

(13) $$APE=\frac{100}{n}\overset{n}{\underset{i=1}{\sum }}\left|\frac{q_{experimental}-q_{predicted}}{q_{experimental}}\right|$$

where *n* is the number of experimental data.

### 2.5. Optical Property Measurements

The classical relation for the near-edge optical absorption of semiconductors was used to analyze the absorption data [38]:

(14) $$\alpha h\nu =B{\left(h\nu -E_{g}\right)}^{m}\;$$

where α is absorption coefficient, *hν* is photon energy, *E_g_* is bandgap, and *B* is constant. In the equation, *m* can have values of 1/2, 2, 3/2, and 3 for allowed direct, allowed indirect, forbidden direct, and forbidden indirect transitions, respectively.

A dependence between the bandgap and wavelength, equivalent to the bandgap energy, *λ_g_,* can be derived [39]:

(15) $$E_{g}=\frac{1239.83}{\lambda_{g}}\;$$

*λ_g_* is determined by extrapolating the linear portion of the curve on the abscissa:

(16) $${\left(\frac{A}{\lambda }\right)}^{1/m}=f\left(\frac{1}{\lambda }\right)\;$$

Because CdTe and CdS are direct bandgap semiconductors, an m value of 0.5 was used.

The absorption coefficient and the photon energy are related by the Urbach energy:

(17) $$\alpha =\alpha_{0}e^{h\nu /E_{U}}\;$$

where *α*_0_ is constant and *E_U_* is Urbach energy. Urbach energy can be calculated using a tangent of the angle of the linear portion of the curve lnA = λ^−1^, according to *E_U_*∙tgθ = 1239.83.

## 3. Results and Discussions

### 3.1. Chalcogens’ and Cadmium Adsorbtions Kinetics

**Figure 2:**
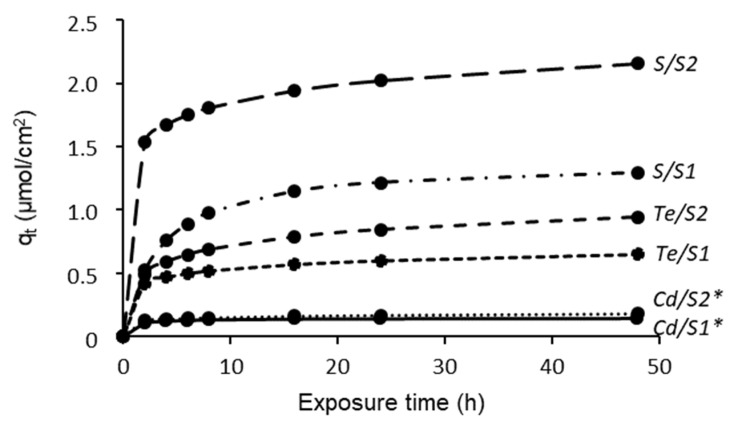
Plots for the sorption of cadmium, tellurium, and sulfur having particles.

The kinetic curves (Figure 2) were determined by changing the initial concentration of the precursor in 0.05 and 0.1 mol/L of the H_2_TeS_4_O_6_ solutions and using an exposure time of 48 h, which was found sufficient to reach saturation. The amount of adsorbed chalcogens containing species depends on precursor concentration and duration of treatment. An increase in the precursor concentration from 0.05 to 0.1 mol/L leads to a S content of approximately 1.3–2.1 µmol/cm^2^ of PA 6 (Figure 2, curves S/S1 and S/S2) and, respectively, the same Te of approximately 0.672–0.934 µmol/cm^2^ of PA 6 (Figure 2, curves Te/S1 and Te/S2) after 48 h of chalcogenization. In addition, the content of S and Te increases with the increase of exposure time. The concentration of S and Te reaches the saturation concentration, respectively, of 2.1 µmol/cm^2^ and 0.934 µmol/cm^2^ after 48 h of chalcogenation at 25 °C.

The cadmium adsorption kinetics on polyamide was investigated when the chalcogens and Cd^2+^ were adsorbed successively. The adsorption curves of Cd on the surface of chalcogenized PA (Figure 2, curves Cd/S1* and Cd/S2*) show that the adsorption of Cd increased as the concentration of chalcogens with species increased. Accordingly, an exposure for 48 h in 0.05 and 0.1 mol/L precursor solution yields a Cd content in PA 6, respectively, of 0.140 µmol/cm^2^ and 0.176 µmol/cm^2^. However, an adsorption of Cd ions on PA is not preferred in comparison with that of chalcogens.

The pseudo second-order rate kinetic model and Elovich‘s equation have been applied to describe the feasibility of chalcogens having species and cadmium sorption onto PA 6, which fitted best the experimental results (minimum APE) (Table 3). The maximum adsorption capacity increased slightly as the concentration increases in all cases. The molar ratio of Cd:Te:S after 48 h of chalcogenization is 1:4.8:9.3 for 0.05 mol/dm^3^ and, respectively, 1:5.3:11.9 for 0.1 mol/dm^3^ initial precursor concentration. The adsorption rate constants, the initial adsorption rate, and the desorption constants were found to increase with decreased concentration of Te and Cd, except for S, where they decreased.

The kinetics’ parameters obtained (e.g., best-fitted kinetic model; the pseudo-second-order rate constants; and the amount of Te, S, and Cd) demonstrate how they may be used in the formation of cadmium chalcogenides’ layers on PA 6 of the desirable phase composition and optical properties.

### 3.2. UV-Vis and FTIR Spectroscopies

**Figure 3:**
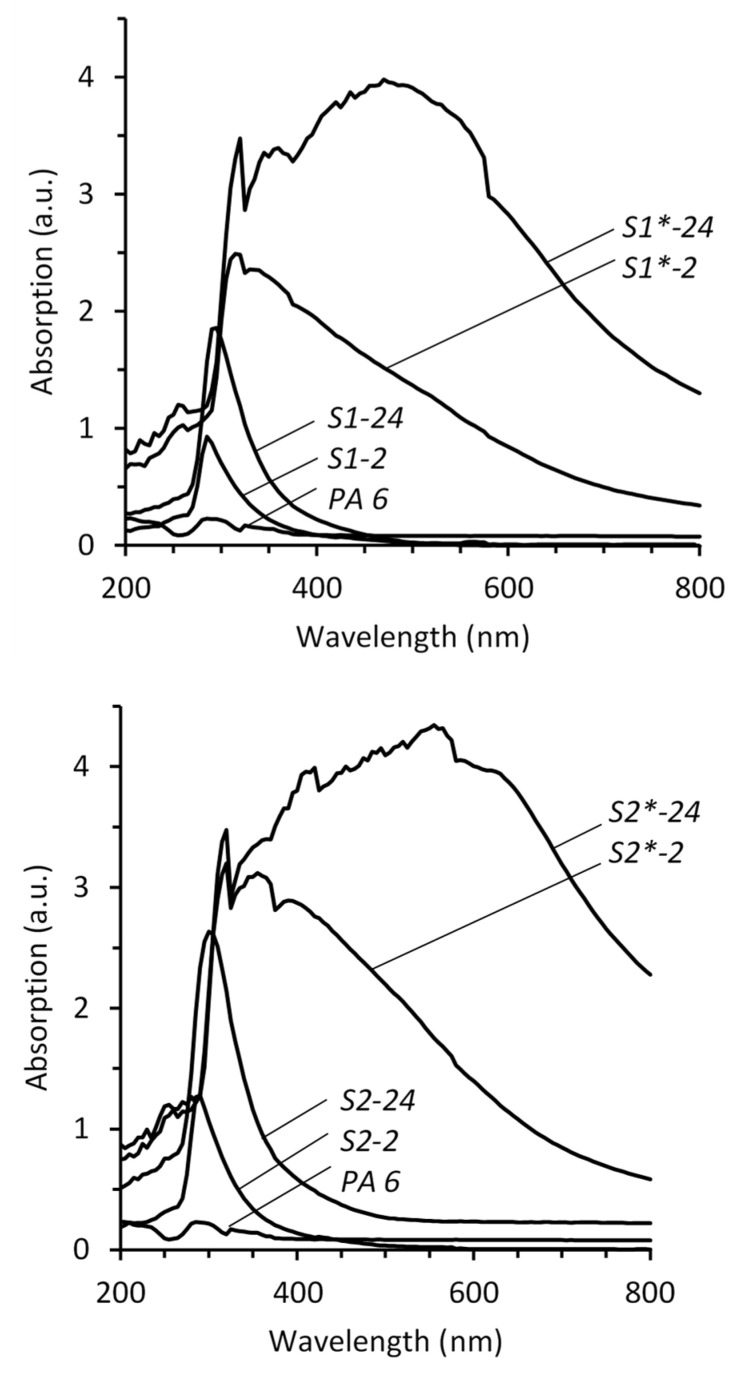
UV-Vis absorption spectra of PA 6, chalcogenized samples in the solutions of H_2_TeS_4_O_6_ and samples with the layers of cadmium chalcogenides.

UV-Vis spectra of chalcogenized PA 6 samples and the spectra of those after treatment in Cd^2+^ salt solution (with compensation of an absorption PA 6) are shown in Figure 3.

In UV-Vis spectra of chalcogenized polyamide, the absorption maxima, were observed at λ = 285–300 nm. Nevertheless, their maximum peak values are shifted towards the shorter wavelengths of the spectrum in comparison to the maximum values of UV-Vis spectra, 310–315 nm of the solutions of H_2_TeS_4_O_6_. All the layers of chalcogens having particles on PA 6 films have very low absorbance near the ultra-violet region of the spectrum (Figure 3, curves S1-2 and S1-24). However, percentage absorption (>90%) of chalcogens having particles containing layers on PA 6 was maximum at λ ~300 nm. With an increase in the exposure time from 2 to 24 h in solutions of the same precursor concentration, the intensity of the maximum of the absorption spectra increased approximately two-fold (Figure 3, curves S1-2 and S1-24, S2-2 and S2-24), and the maximum peaks are shifted towards the longer wavelength region. The intensity of the spectrum of PA 6 treated in 0.05 mol/L. H_2_TeS_4_O_6_ varies from 0.928 to 1.859 absorption units (a.u.) and, respectively, from 1.277 to 2.635 a.u. of the same treated in 0.1 mol/L H_2_TeS_4_O_6_. The concentration of the precursor also increases the effect of increasing absorption at the maximum wavelength; the clear shift to the direction of longer wavelength, e.g., 290–300 nm, was observed in the spectra of PA samples treated in 0.1 mol/L H_2_TeS_4_O_6_ in comparison with the wavelength region 285–295 nm of the spectra of the PA samples treated in 0.05 mol/L H_2_TeS_4_O_6_. A variety of new peaks occurs in the range 315 to 600 nm in the layers with cadmium chalcogenides compared to peaks before interaction with Cd^2+^ (Figure 3, curves S1*-2 and S1*-24, S2*-2 and S2*-24). These new broad and unclear peaks occur in the spectra of PA chalcogenized in the 0.05 mol/L H_2_TeS_4_O_6_ solution at 315–470 nm and, respectively, at 320–560 nm after chalogenization in 0.1 mol/L H_2_TeS_4_O_6_. Only the peaks in the 315–325 nm regions are sharper (Figure 3, curves S1*-2 and S1*-24, S2*-2 and S2*-24). These peaks are higher than the corresponding peaks of PA 6 before the interaction with cadmium, indicating the existence of cadmium chalcogenides. To describe the formation of cadmium chalcogenide in general terms, the formula CdTe–CdS is used. It is assumed that these new peaks show cadmium telluride or mixed cadmium telluride-sulfide, which occurred after treating with Cd^2+^. Some experiments are needed to clarify the attribution of the new UV-Vis absorption bands. Some of the methods applied provide information, but the data interpretation on the PA surface is complicated. The next experiment will focus on a mechanism in an aqueous solution and is explained in reactions on polyamide.

The spectra of optical absorption of the chalcogens species having layers on PA 6 showed that they have high absorbance (≥10^4^ cm^−1^) in the UV-Vis region, with a gradual decrease towards the NIR region of the electromagnetic spectrum indicating direct bandgap transition.

**Figure 4:**
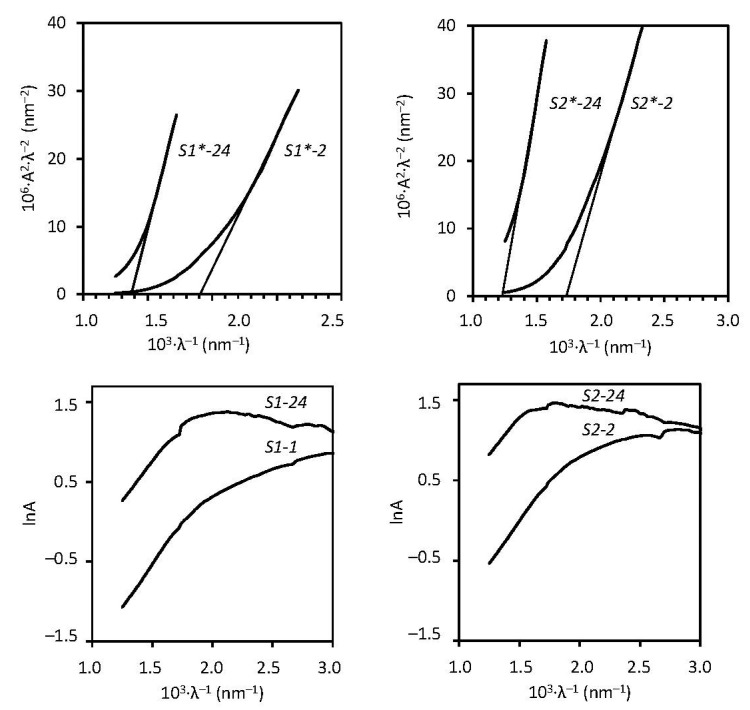
Tauc plots (**top**) and Urbach plots (**bottom**) of cadmium chalcogenide layers on PA 6.

According to literature data [40] the bandgap values are between 1.517 eV (CdTe) and 2.41 eV (CdS); see Table 4. The bandgap values of cadmium chalcogenide layers with different stoichiometry were as follows: with a longer chalcogenization time in 0.05 mol/L H_2_TeS_4_O_6_, the bandgap decreases from 2.39 eV to 1.70 eV, and from 2.15 eV to 1.52 eV in 0.1 mol/L H_2_TeS_4_O_6_, respectively (Figure 4, Table 5). The determined bandgap values were higher when compared to the bulk values (Table 4). This shows that mixed CdTe–CdS chalcogenides are formed. Tellurium as a metaloid has a very low bandgap value (approximately 0.33 eV) [40]. Even though diffractograms did not detect elemental tellurium, the molar ratio of Cd: Te: S in the layers shows there should be amorphous tellurium. This determines the decrease of the bandgap value of the layers when the concentration of the solution of the precursor and exposure time increases. At higher saturation of the PA 6 with cadmium and chalcogens species, a redshift of the absorption is observed in the spectra of the samples, which results in lower bandgap values. This is also due to the occurrence of an increased amount of the amorphous phase (Te) mixed with the nanocrystalline phase. Urbach energy changes from 0.56 eV to 0.62 eV with the increased treatment time in 0.05 mol/L H_2_TeS_4_O_6_ and from 0.58 eV to 0.66 eV in 0.1 mol/L H_2_TeS_4_O_6_, respectively (Figure 4, Table 5). The Urbach energy shows the density of states at the edge of the band and the local microstructural disorder. Therefore, the lower Urbach energy indicates fewer defects in structural bonds.

The mixed layers of cadmium chalcogenides should have more applications because its optical energy gap could be tuned by means of the composition between 1.52 and 2.36 eV.

**Figure 5:**
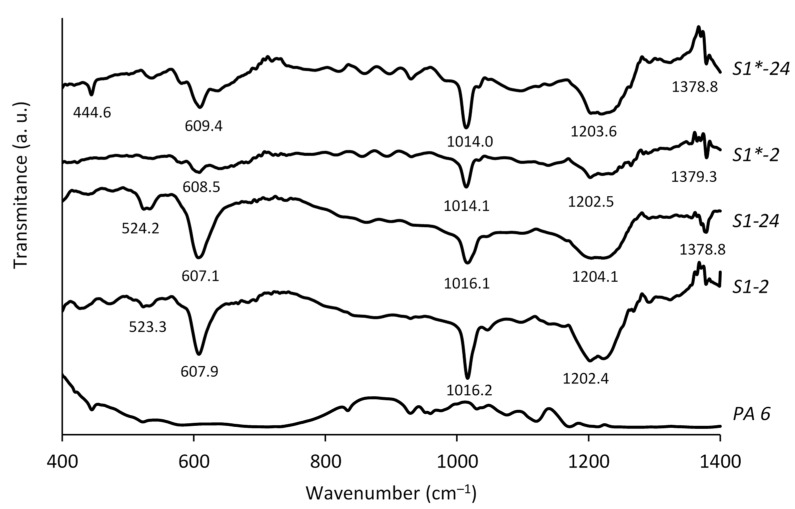

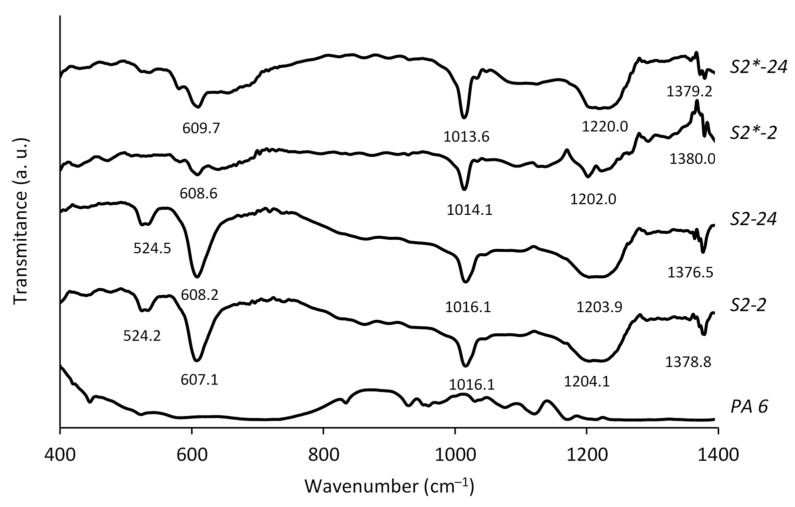
FTIR absorption spectra of PA 6 chalcogenized samples in the solutions of H_2_TeS_4_O_6_ and samples with the layers of cadmium chalcogenides on the PA surface.

From the FTIR spectra in the region of frequencies 500–1300 cm^−1^ of the polyamide surface before and after chalcogenization with monotelluropentathionic acid, it can be seen that the new material was formed or attached to the polyamide surface after chalcogenization (Figure 5). In the ranges of wave numbers 523–608 cm^−1^ and 1016–1204 cm^−1^, four peaks of the most intense bands are seen in the spectra of chalcogenized PA 6. S_2_O_3_^2−^ vibration can be assigned to six main absorption bands in the area of 520–1200 cm^−1^ [41]: for asymmetric deformation vibration, δ_as_ (O–S–O), for symmetric deformation vibration, δ_s_(O–S–O), for symmetric valent, υ_s_(S–O), and for asymmetric valent, υ_as_(S–O).

Cadmium chalcogenide layers exhibited different IR spectra compared to an only-chalcogenized PA sample (Figure 5, curves S1-2 and S1-24, S2-2 and S2-24). As we can see, in the frequency region from 605 to 610 cm^−1^, the absorption of infrared light decreases, but it increases in the regions at 1013–1016 cm^−1^ and at 1203–1204 cm^−1^. Meanwhile, in the cases of increased PA 6 chalcogenization time and precursor concentration, the absorption of infrared light increased. Additionally, new peaks as shoulders in the region at 750–950 cm^−1^ appeared.

The broad band is shown with a maximum in the range 1209–1215 cm^−1^. The absorption at lower frequencies, with a peak at 609 cm^−1^, was strong enough, and the band was sharp and distinct so that it was combined with valence absorption. The band at 608–610 cm^−1^ can be assigned to the symmetric deformation O–S–O vibrations, δ_s_(O–S–O), in the terminal SO_3_ groups of the polythionates [31].

The absorption peaks in the 790–1302 cm^−1^ region can be attributed to the asymmetric valence S–O vibrations, ν_as_(S–O), and the symmetric valence S–O vibrations, ν_s_(S–O), in the terminal SO_3_ groups of polythionates. [42].

However, the absorption intensity from 524 to 735 cm^−1^ increases significantly as chalcogenization proceeds. The bands with absorption peaks in this area correspond to symmetric deformation of O–S–O vibrations, δ_s_(O–S–O), and asymmetric deformation vibrations of O–S–O, δ_as_(O–S–O), in the terminal SO_3_ groups of polythionates [41].

The FTIR spectra propose only the probable course of the formation of thin chalcogen layers.

For the formation of nanolayers on the surface of PA, the main process is that the obtained layers of chalcogens having species, possibly chalcogenides, block the polymer surface, changing their properties. It can be assumed that new groups have formed, which are polychalcogenides and polychalcogenes. Further treatment with a cadmium salt solution leads to the formation of mixed CdTe–CdS layers, the FTIR spectra of which differ significantly from the spectra of chalcogenized PA 6 (Figure 5).

Some additional experiments are needed to determine the structural affiliation of the FTIR absorption bands. The transition metal chalcogenides have been extensively studied for their rich structural chemistry [43,44]. These compounds exhibit weak van der Waals interactions within a layer or chain, but a strong chalcogen-metal bond between these layers or chains. The chalcogen–metal bonds in transition metal chalcogenides are more covalent; therefore, chalcogen ions are less charged. The reduced electronegativity from S to Te is frequently accompanied by Q–Q bonding [45]. Different structural and physical properties could be related to the large ionic radius and reduced electronegativity.

### 3.3. XRD Characterization and the Mechanism of Cadmium Chalcogenides Layers Formation on the Substrate of Polyamide 6 Film

The structural characterization of the obtained CdTe–CdS layers was carried out using XRD. Due to the polycrystalline nature of the formed CdTe–CdS, as well as the simultaneous existence of many phases of cadmium telluride, cadmium sulfide, and sulfur of various chemical compositions and a high degree of crystallinity of the polyamide itself, complex diffraction patterns were obtained. The intensity of cadmium chalogenide peaks is several times lower than that of PA 6 peaks at 2θ < 28°. Thus, only the 30–70° region was used in detail in combination with JCPDS reference patterns and the available literature data [46,47,48,49].

**Figure 6:**
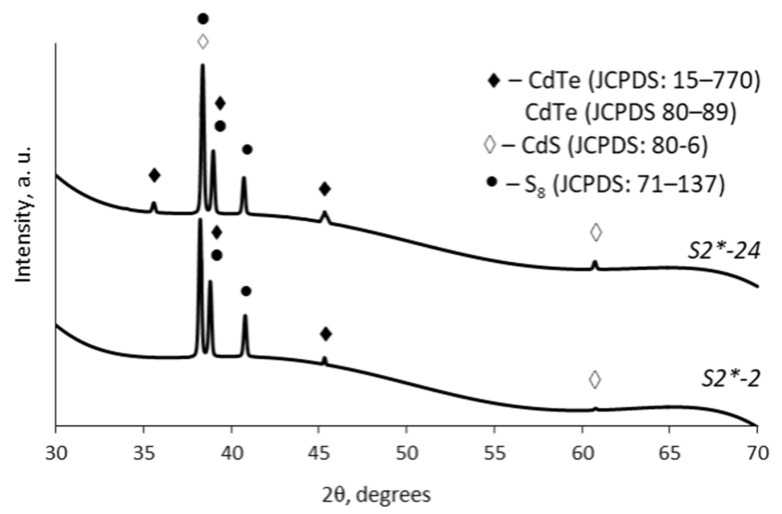
X-ray diffraction patterns of the layers of cadmium chalcogenides on PA 6 films surface.

Selected XRD diffractograms of cadmium chalcogenide layers on PA 6 film are shown in Figure 6, and the corresponding peak values are presented in Table 6. It can be seen that the cadmium chalcogenide layers consist of three phases: hexagonal cadmium telluride (CdTe, JCPDS card No 80-89), cubic cadmium telluride (CdTe, JCPDS card No 15-770), and hexagonal (CdS, JCPDS card No 80-6)) and monoclinic sulphur (S_8_, JCPDS card No 71-137). It can also be observed that as the chalcogenization time and H_2_TeS_2_O_6_ concentration increases, the amount of the mentioned phases in the cadmium chalcogenide layer also increases.

Because the details of the mechanism for both stages of treatment have not yet been elucidated, the proposed tentative mechanism is as follows.

At the first stage, TeS_4_O_6_^2−^ anions adsorb and diffuse into the surface of PA 6. In the second stage, chalcogenized samples reacted with cadmium sulfate solution and the formation of cadmium chalcogenide layer proceeds via [Cd(H_2_O)_2_(NH_3_)_4_]^2+^ ion reactions with the adsorbed TeS_4_O_6_^2−^ ion according to reactions (18)–(21):

2TeS_4_O_6_^2−^ + 5[Cd(H_2_O)_2_(NH_3_)_4_]^2+^ + 16OH^−^ → 2CdTe + 3CdS + 5SO_4_^2−^ + 18H_2_O + 20NH_3_ (18)

The fact that elemental sulfur was found in the cadmium chalcogenide layer can be explained by the fact that the anions of TeS_4_O_6_^2−^ in the surface of PA 6 are unstable at 80 °C. A part of them does not react with [Cd(H_2_O)_2_(NH_3_)_4_]^2+^ ions during the next step and decompose according to the reaction (19):

TeS_4_O_6_^2−^ + 4OH^−^ → 2S + 2SO_4_^2−^ + Te^2−^ + 2H_2_O (19)

The anions of SO_4_^2−^ wash out, and elemental sulfur remain in the polymer. Meanwhile, the anions of Te^2−^ react with [Cd(H_2_O)_2_(NH_3_)_4_]^2+^ ions:

[Cd(H_2_O)_2_(NH_3_)_4_]^2+^ + Te^2−^ → CdTe + 4NH_3_ + 2H_2_O (20)

The total equation of the second step is:

3TeS_4_O_6_^2−^ + 6[Cd(H_2_O)_2_(NH_3_)_4_]^2+^ + 20OH^−^ → 3CdTe + 3CdS + 2S + 7SO_4_^2−^ + 22H_2_O + 24NH_3_ (21)

The procedures for attaching functionalized chelating polymers to the inner pore surface for the sorption of metal ions have been extensively studied [50,51]. A special role must also be attributed to an interaction of cadmium Cd^2+^ ions with the sorbed products from the chalcopolythionate anions. Sulfur or tellurium, either as free chalcogenide ions or when covalently bound to the amido group of PA, behave as a very effective Lewis base with respect to many transition metals, e.g., copper, iron, molybdenum, or zinc [52].

### 3.4. Morphological Characterization of Cadmium Chalcogenide Layers

**Figure 7:**
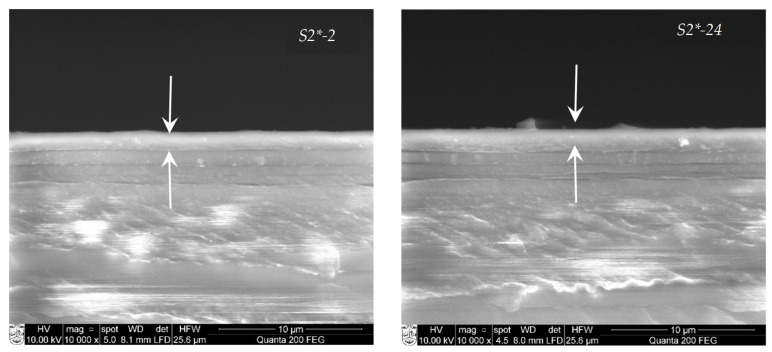
SEM images of cross-sections of cadmium chalcogenide layers on PA 6.

SEM is a convenient technique for the investigation of cross-sections of PA 6 samples with cadmium chalcogenide layers. The SEM images for the cadmium chalcogenide layers on PA 6 show that the layers appear as homogeneous and dark strips, and their thickness depends on the time of the chalcogenization. The thickness of cadmium chalcogenide layers increases from 1.3 ± 0.1 μm to 1.7 ± 0.15 μm with the increase in the time of the chalcogenization from 2 to 24 h. The cross-sections (Figure 7) show that there is a good adherence of the layer to the polymer substrate.

**Figure 8:**
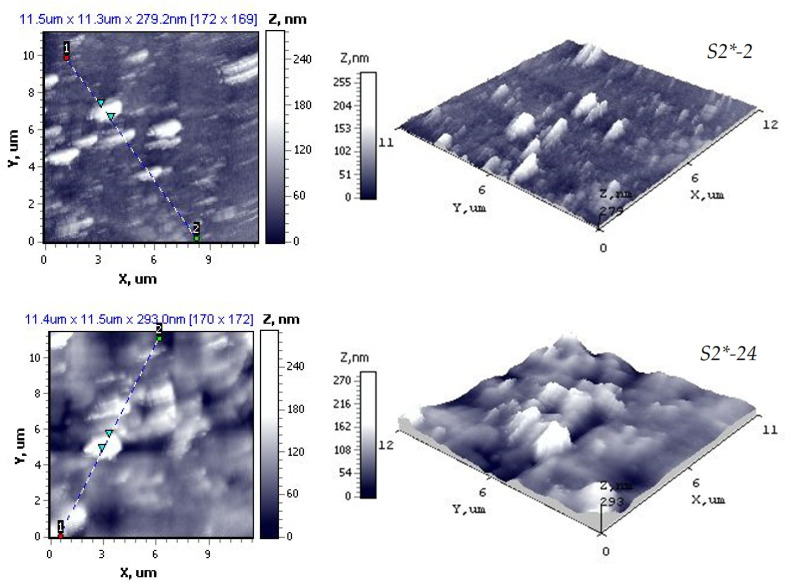
AFM images of cadmium chalcogenide layers on PA 6.

The SEM micrograph shows a compact polycrystalline surface, consisting of one type of small, densely packed grains, unevenly distributed over a smooth homogeneous background. A small amount of compositional variation is observed in the micrographs of the deposited cadmium chalcogenide layers due to the longer exposure time in the precursor solution. A more detailed morphology was investigated by AFM, and the results are presented in Figure 8 and Table 7.

Figure 8 shows the top view 11.6 × 11.4 μm and 11.5 × 11.6 μm images of cadmium chalcogenides layers after 2 h (S2*-2) and 24 h (S2*-24) of chalcogenization, respectively. The surface images show (Figure 8) that the grains are unevenly distributed on the surface of the layers. Therefore, the interconnected grain particles merged together to form grains with an average diameter of ~30 nm. An agglomeration of grains takes place in the chalcogenide layers formed after a longer chalcogenization. The roughness parameters (Table 7) were determined using the section from the upper right position to the lower left of the top view image. RMS values of 29.97–35.21 nm at 279.2–293.0 nm maximum height allows determination of the layers of cadmium chalcogenides on PA 6 and demonstrate their sufficient high surface characteristics.

It is necessary to mention that mixed cadmium chalcogenide layers formed on PA 6 have specific compositional and morphological properties different from bulk chalcogenides, e.g., CdS or CdTe. The regularities established for the formation of those layers allow modulation in the formation of layers with desirable composition and morphology. Very simple procedures are required—the correct selection of single-source binary precursor, precursor concentration, and treatment time. The tunable bandgap is characteristic of these mixed binary layers of cadmium chalcogenides.

Such semiconductors of varying conductivity and bandgap values close to the solar spectrum are potential candidates for the fabrication of thin-film solar cells.

## 4. Conclusions

The successive ionic layer adsorption and reaction (SILAR) method was used to obtain ~1.3–1.7 μm cadmium telluride–cadmium sulfide layers on the surface of polyamide 6, and their optical, morphological, and crystalline phase information was determined. To obtain these layers, H_2_TeS_2_O_6_ 0.05 and 0.1 mol/L solutions at 25 °C and alkali solution (pH 11.5) of 0.1 mol/L divalent cadmium in the complex ions form, [Cd(H_2_O)_2_(NH_3_)_4_]^2+^, at 80 °C were used. The quantity of Te, S, and Cd in the formed layers increases with increasing concentrations of chalcogens precursor solution and exposure time from 2 h to 48 h, respectively for Te ~0.672–0.934 µmol/cm^2^, for S ~1.3–2.1 µmol/cm^2^, and for Cd ~0.140–0.176 µmol/cm^2^. It was found that the initial rate of adsorption and the rate constants of adsorption and desorption increase with decreasing concentration of Te and Cd in the layers, except for S, where they decreased. The UV-Vis absorption spectra of the chalcogenides on PA 6 showed that the layers have high absorbance (≥10^4^ cm^−1^), indicating direct bandgap transition. The optical study reveals that the direct transition bandgap for cadmium chalcogenide layers with different stoichiometry is found to be 1.70 to 2.36 eV for the samples chalcogenized in 0.05 mol/L solution of H_2_TeS_4_O_6_ and 1.52 to 2.15 eV for the samples chalcogenized in 0.1 mol/L solution. FTIR spectra of the modified polyamide show that, after chalcogenization, the new material is chemically bonded to the PA functional groups. XRD analysis of the cadmium chalcogenide layers on the PA 6 film showed that they consist of phases: hexagonal and cubic cadmium telluride, hexagonal cadmium sulfide, and monoclinic sulfur. The AFM and SEM studies showed that the mix layers of cadmium telluride and cadmium sulfide consist of crystalline grains with an average diameter of approximately 30 nm, some of which are agglomerated. Lower surface roughness parameters and a significantly smoother surface are observed because of shorter exposure time in the solution of the precursor. This correlates well with the calculated Urbach energy values, which show a lower microstructural disorder of the sample treated in the precursor solution for 2 h.

## Figures and Tables

**Table 1:** The main characteristics of Polyamide 6.

Property	Dry/Moist	Unit
Tensile strength at yield	85/60	MPa
Elongation at yield	4	%
Elongation at break	70/200	%
Hardness	160/70	N/mm^2^
Glass transition temperature	60/5	°C
Thermal conductivity (23 °C)	0.23	W/(K·m)
Density	1.13	g/cm^3^
Moisture absorption (23 °C)	3	%
Water absorption to equilibrium	9.5	%

**Table 2:** Duration of chalcogenization of PA 6 films at 25 °C and sample labelling.

Duration, h	Concentration of H_2_TeS_4_O_6_, mol/L	
	0.05	0.1
	Sample No	
2	S1-2	S2-2
4	S1-4	S2-4
6	S1-6	S2-6
8	S1-8	S2-8
16	S1-16	S2-16
24	S1-24	S2-24
48	S1-48	S2-48

The sign “*” was added for the labeling of chalcogenized PA samples after their treatment with a cadmium salt solution (S1* and S2*).

**Table 3:** Model parameters for tellurium, sulfur, and cadmium species sorption kinetics by PA 6.

Model	Parameters	Tellurium		Sulfur		Cadmium	
		H_2_TeS_4_O_6_ (mol/L)					
		0.05	0.10	0.05	0.10	0.05	0.10
Lagergren	*r* ^2^	0.7786	0.9026	0.9265	0.9044	0.9843	0.8635
	*q_e_* (μmol/cm^2^)					0.087	
	*k*_1_ (h^−1^)					0.261	
	APE (%)					46.7	
Ho	*r* ^2^	0.9961	0.9989	0.9996	0.9998	0.9999	0.9999
	*q_e_* (μmol/cm^2^)	0.691	0.993	1.382	2.148	0.143	0.182
	*k*_2_ (cm^2^·μmol^−1^·h^−1^)	0.618	0.290	0.218	0.406	8.602	3.312
	ARE (%)	8.1	3.1	1.7	2.6	1.6	1.7
Elovich	*r* ^2^	0.9825	0.997	0.9622	0.9651	0.853	0.9798
	*a* (μmol·cm^−2^·h^−1^)	11.601	2.247	1.491	263.53		9.967
	*b* (cm^2^·μmol^−1^)	13.850	7.057	4.198	5.133		57.471
	APE (%)	2.1	0.9	4.9	1.9		1.3
Boyd	*r* ^2^	0.3757	0.762	0.6927	0.7381	0.9595	0.7536
	R (h^−1^)					0.289	
	APE (%)					11.6	
Weber–Morris	*r* ^2^	0.3537	0.6051	0.621	0.1671	0.122	0.4035
	*k_int_* (μmol·cm^−2^·h^−1/2^)						
	APE (%)						
Dumwald–Wagner	*r* ^2^	0.7128	0.9458	0.9641	0.9045	0.9954	0.8996
	*K* (h^−1^)			0.084		0.250	
	APE (%)			4.8		3.1	

**Table 4:** Bandgap values of CdS and CdTe compounds [40].

Compound	CdS	CdTe	
Bandgap, eV	2.41–2.425	1.517	1.58
Temperature, K	300	300	300
method of determination	photoconduction	electroreflection	thermal activation
type of sample	single crystalline	single crystalline	single crystalline
transition	direct allowed	direct	direct allowed

**Table 5:** Optical properties of cadmium chalcogenide layers.

Sample No	Molar Ratio Cd:Te:S	Direct bandgap *E_g_* (eV)	*R* ^2^	Urbach Energy (eV)	*R* ^2^
S1*–2	1:4.3:5.4	2.36	0.9973	0.56	0.9993
S1*–24	1:4.3:8.6	1.70	0.9995	0.62	0.9998
S2*–2	1:5.2:13.0	2.15	0.9993	0.58	0.9997
S2*–24	1:5.1:12.2	1.52	0.9946	0.66	0.9994

**Table 6:** XRD peak assignments of cadmium chalcogenide layers on PA 6.

Sample No				CdTe				CdS (JCPDS 80–6)		S_8_ (JCPDS 71–137)	
S2*-2		S2*-24		(JCPDS 80–89)		(JCPDS 15–770)					
2θ	d (Å)	2θ	d (Å)	d (Å)	h k l	d (Å)	h k l	d (Å)	h k l	d (Å)	h k l
		35.618	2.519	2.524	1 0 3						
38.444	2.340	38.444	2.340					2.439	1 0 2	2.341	1 4 2
39.093	2.302	39.093	2.302			2.29	2 2 0			2.304	1 2 4
40.789	2.210	40.789	2.210							2.213	3 2 3
45.331	1.999	45.331	1.999	1.991	1 1 2	1.954	3 1 1				
60.752	1.523	60.752	1.523					1.513	1 0 4		

**Table 7:** Surface roughness parameters of cadmium chalcogenide layers chalcogenized in 0.1 mol/L solution of H_2_TeS_4_O_6_ at 25 °C.

Sample No	Max. Height, A (nm)	Average Height, Z_mean_ (nm)	Average Roughness, R_a_ (nm)	RMS Roughness, R_q_ (nm)	Skewness, R_sk_ (nm)
Crude sample	67.4	27.76	3.45	4.59	0.82
S2*-2	279.2	62.04	20.09	29.97	1.26
S2*-24	293.0	92.72	25.87	35.21	0.92

## Data Availability

Not applicable.
